# Ameliorative Effect of Graviola (*Annona muricata*) on Mono Sodium Glutamate-Induced Hepatic Injury in Rats: Antioxidant, Apoptotic, Anti-inflammatory, Lipogenesis Markers, and Histopathological Studies

**DOI:** 10.3390/ani10111996

**Published:** 2020-10-30

**Authors:** Mustafa Shukry, Ahmed M. El-Shehawi, Wafaa M. El-Kholy, Rasha A. Elsisy, Hazem S. Hamoda, Hossam G. Tohamy, Mohamed M. Abumandour, Foad A. Farrag

**Affiliations:** 1 Department of Physiology, Faculty of Veterinary Medicine, Kafrelsheikh University, 33511 Kafrelsheikh, Egypt; 2 Department of Biotechnology, College of Science, Taif University, P.O. Box 11099, Taif 21944, Saudi Arabia; elshehawi@hotmail.com; 3 Department of Genetics, Faculty of Agriculture, Alexandria University, 21527 Alexandria, Egypt; 4 Department of Zoology, Faculty of Science, Mansoura University, 35516 Mansoura, Egypt; wafaa_elkholy2002@yahoo.com; 5 Department of Anatomy, Faculty of Medicine, Kafrelsheikh University, 33516 Kafrelsheikh, Egypt; Rasha_2002@yahoo.com; 6 Department of Anatomy and Embryology, Faculty of Veterinary Medicine, Aswan University, 81528 Aswan, Egypt; Hamouda2000@yahoo.com; 7 Department of Pathology, Faculty of Veterinary Medicine, Alexandria University, 22785 Alexandria, Egypt; hossam.gafar@yahoo.com; 8 Department of Anatomy and Embryology, Faculty of Veterinary Medicine, Alexandria University, 22785 Alexandria, Egypt; m.abumandour@yahoo.com; 9 Department of Anatomy and Embryology, Faculty of Veterinary Medicine, Kafrelsheikh University, 33511 Kafrelsheikh, Egypt; foad.farrag@yahoo.com

**Keywords:** Graviola, liver, monosodium glutamate, antioxidant, apoptosis, iNOS, SIRT1

## Abstract

**Simple Summary:**

Food additives, especially monosodium glutamate (MSG), induces serious liver disorders. This study premeditated to investigate the effect of Graviola extract (GE) on hepatic and cellular alterations induced by MSG. Our result revealed that GE administration normalized the oxidative stress markers, as well as the proinflammatory cytokines, in addition to downregulation of the inducible nitric oxide synthase (iNOS) and FAS, hepatic fatty acid synthase, and led to the upregulation of the silent information regulator protein one (SIRT1) gene. This is the first report investigating the intracellular pathway and mechanism of Graviola extract’s action in alleviating the MSG supplementation injuries.

**Abstract:**

Monosodium glutamate (MSG) is a widely used food additive, and there is a trepidation that MSG plays a critical role in multiple hepatic disorders. This study was planned to investigate Graviola extract (GE) effects on hepatic and cellular alterations induced by MSG. Fifty Wistar rats were randomly allocated into five groups: control (received normal saline), Graviola (received 200 mg/kg body weight), MSG (received 2.4 gm MSG/kg, 15% of Lethal dose (LD_50_) of MSG), Graviola + monosodium glutamate (MSG + GE; received GE, 200 mg/kg/day and MSG 2.4 gm/kg body weight (BW) for the next four weeks), and monosodium glutamate + Graviola (received MSG only (2.4 gm/kg BW) daily for four weeks, then concomitant with Graviola (200 mg/kg BW) daily for the next four weeks. MSG and GR were administered orally for eight weeks. Our results showed that MSG caused a significant increase in oxidative stress markers malondialdehyde (MDA), reactive oxygen species (ROS), nitric oxide (NO), hydrogen peroxide **(**H_2_O_2_**)**, proinflammatory cytokines interleukin 6 (IL-6) level, a tumor protein (P53), hepatic cellular damage, as well as proapoptotic markers caspase-3, and B-cell lymphoma 2 (BCL-2)-like protein 4 (Bax). A significant decrease in superoxide dismutase (SOD), catalase (CAT), glutathione S transferase (GST), reduced glutathione (GSH), and an antiapoptotic agent B-cell lymphoma 2 (BCl-2) was observed. The detected MSG effects were normalized by Graviola administration, either a prophylactic or protecting dose. Besides, Graviola reduced the expression of inducible nitric oxide synthase (iNOS) and hepatic fatty acid synthase (FAS) and led to the upregulation of the silent information regulator protein one gene expression gene (SIRT1).In conclusion, the results suggest that Gaviola’s interrelated antiapoptotic, antioxidant, and anti-inflammatory properties are potential mechanisms to enhance hepatic deficits and protect the liver. Graviola can, therefore, be considered a promising hepatoprotective supplement. Additionally, further human clinical trials are also necessary to validate the present research.

## 1. Introduction

Many synthetic contaminants, such as industrial toxins and food additives, have been implicated in adverse effects. Some food additives act as either preservatives or palatability enhancers. One such food additive is monosodium glutamate (MSG). MSG is one of these food additives that is openly used as a flavor enhancer. It is glutamic acid salt [1]. It is documented that rats administrated MSG encountered many disorders such as gonadal dysfunction, an increase in stomach cancer incidence, brain damage, learning difficulty, and depletion in some neurotransmitters in the hypothalamus region [2]. The extravagant administration of MSG was shown to cause liver and kidney damage [3], as well as oxidative stress in the tissue, with degenerative changes in hepatocytes [4]. MSG enhances meals’ palatability and significantly improves the appetite center and, consequently, increases body weight [5]. Although MSG enhances flavor stimulation and boosts appetite, it is considered toxic to humans and experimental animals [6]. In normal conditions, glutamate has a very low acute toxicity; the oral dose lethal to LD_50_ in rats and mice is ∼15,000–18,000 mg/kg body weight, respectively [7]. Besides, an oral gavage dose of MSG for a dose level of 0.8, 1.6, and 2.4 gm/kg body weight (BW)/day for 30 and 40 days, respectively, presumably suppresses the female’s reproductive system in rats [8]. In the intoxicated model, MSG rats, a daily dose of MSG (4 g/kg orally) was given for seven days, which induced kidney injury in rats [9]. According to the last update of the Joint Food and agriculture organization (FAO)/WHO Expert Committee on Food Additives (JECFA), the U.S. Food and Drug Administration (FDA), and the European Food Safety Association (EFSA), the acceptable daily intake of MSG to humans is 30 mg/kg/day [10]. The acceptable daily intake (ADI) is the maximum amount of a chemical that can be ingested daily over a lifetime with no appreciable health risk and is based on the highest intake that does not give rise to observable adverse effects.

The liver is the body’s most prominent glandular tissue, and it has a significant role in body metabolism. It has a wide range of functions, including the storage of glucose, plasma protein synthesis, and bile production [11]. The liver may be susceptible to harm due to toxic substances, because it is involved in these diversified functions. Appropriate alternative therapies should be sought to increase the treatment’s efficacy by recognizing the pathophysiological processes responsible for creating hepatic injury. The disparity between the generation of ROS and antioxidants leads to free radical species disruption to oxidative substances in cells, including proteins, lipids, or nucleic acids [12]. Supplementations with natural antioxidants have been shown to improve the body’s efficiency in stressful conditions [13]. Natural medicines have traditionally been used to cure several diseases. Muricata Annona L. (Graviola), a member of the Annonaceae family, is a perennial tree species used as herbal medicine. The plant has various pharmacological functions, including cytotoxicity, antimicrobial, and wound care [14]. It has antidiabetic and hypolipidemic [15], antiarthritic [16], antinociceptive [17], hepatoprotective and bilirubin-lowering [18], powerful antioxidants activities [19], antihypertensive [20], anticancer [21], gastroprotective [22], and anti-inflammatory and anticonvulsant [23] effects. The most useful aspects of this tree are Graviola leaves. It has active ingredients, bulatacin, asimisin, and squamosin, which contain acetogenins [24]. The presence of secondary metabolites like tannins, steroids, and cardiac glycoses was revealed by a chemical screening of Graviola extract [25]. The current research was carried out to study Graviola’s prophylactic and protective effects on rats’ MSG-induced hepatic injuries.

## 2. Materials and Methods

### 2.1. Chemicals

All chemicals and kits used were purchased from standard confirmed companies and were of the highest grade. MSG (<99%) was purchased from Sigma-Aldrich Co. (St. Louis, MO, USA). Complete RNA extraction and SYBR Green Master Mix kits were purchased from (QIAGEN, Hilden, Germany). Graviola dry extract^®^ was imported from Origini Naturali Company (Quarrata, Pistoia, Italy).

### 2.2. Ethics Statement

The study was approved with the NIH Guide for the Treatment and Use of Animals from the University of Kafrelsheikh, Egypt, Faculty of Veterinary Ethics Committee. All procedures were taken during the process to alleviate animal suffering. Egypt KSU/VetMed-2018-/1155.

### 2.3. Experimental Design

**Figure 1:**
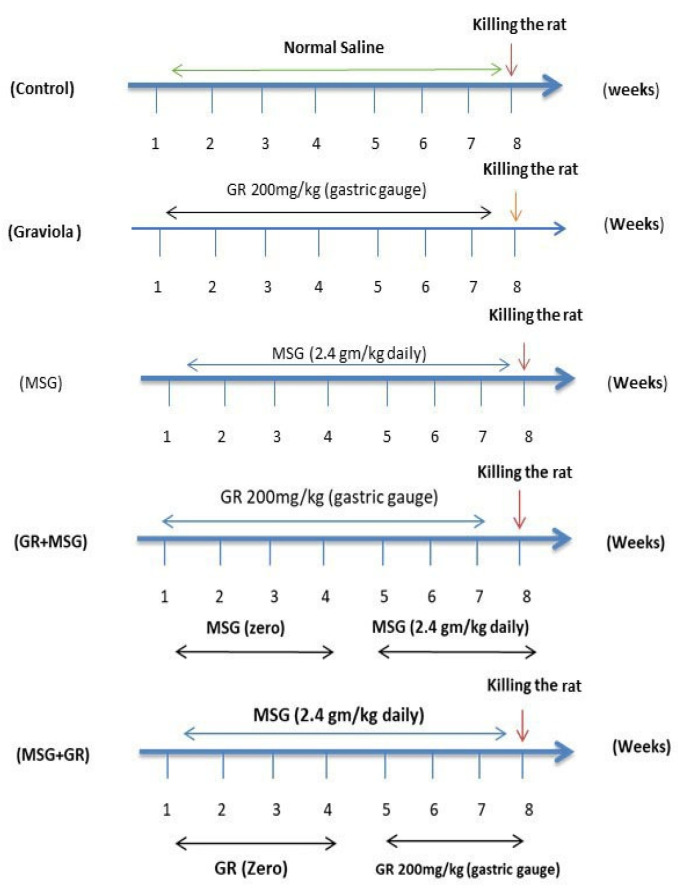
**** Time scheme of the experiment. MSG: monosodium glutamate. GR: Graviola.

Fifty Wistar male rats ten weeks of age average (179 ± 1 g) were purchased from the Egyptian Institute for Vaccine and Serological Production, Helwan, Egypt and were housed in the animal house of the Department of Physiology, Faculty of Veterinary, Kafrelsheikh University. They were maintained under standard laboratory conditions with a 12 h light/dark cycle and free access to food pellets and tap water ad libitum. After two weeks of acclimatization, the rats were randomly allocated into five groups as seen in Figure 1: control (received normal saline (0.9%) with the same procedure and volume as Graviola-treated groups, orally once daily for eight weeks), Graviola (orally administrated Graviola extract (GE) at 200 mg/kg BW daily for 8 weeks), and Graviola dry extract was dissolved in normal saline (0.9%) and administered daily with 200 mg extract/kg BW using gavage needles. The selected dose of aqueous Graviola extract (GE) was according to the previous study of [26,27], MSG (orally administrated 15% of LD_50_ of MSG, 2.4 gm/kg daily for 8 weeks) according to [8], Graviola + monosodium glutamate (MSG + GR; received GE, 200 mg/kg/day once daily orally four weeks, then concomitant with MSG 2.4 gm/kg BW for next four weeks), and monosodium glutamate + Graviola (orally administered MSG only (2.4 gm/kg BW) daily for four weeks, then concomitant with GE (200 mg/kg BW) daily for next four weeks. All treatments were initiated and continued for eight weeks with a basal diet [28,29]. The body weight and food intake were daily recorded.

### 2.4. Sampling

Rats at the end of the experimental period (eight weeks) were anesthetized and sacrificed for sampling and consequent analysis by intravenous sodium pentobarbital injection (30 mg/kg). Blood samples were collected part in centrifuge clean glass tubes, left to clot, and centrifuged at 4000 rpm for 15 min. The clear, not hemolyzed sera were quickly removed and put in labeled Eppendorf tubes; the sera were frozen at −20 °C for further biochemical analysis. The other part was collected in sterilized Ethylenediaminetetraacetic acid (EDTA) tubes (Al-Gomhuria Chemical Company, Cairo, Egypt) for hematological analysis. Liver samples (n = 6 per group) from each rat were removed, weighed, and homogenized in cold phosphate-buffered saline (PBS). At 4 °C, the homogenates were centrifuged for 10 min at 3000× *g*. For biochemical assays, the collected supernatants were stored at −20 °C. The remaining liver specimen was held frozen at −80 °C to study gene expression and other biochemical assays. Different samples of the liver tissue were stored in neutral formalin (10%) for histopathological studies.

### 2.5. Biochemical and Hematological Analysis

Materials and services in this section were obtained from Bio-Diagnostic Co. Dokki, Giza, Egypt, unless otherwise indicated. The automatic measurement of hematology parameters was performed using an Avantor Performance Materials Inc. Business, Center Valley (USA), H32 VET 3-Part differential analyzer of hematology. Serum total cholesterol and triglyceride concentration were estimated according to the method of [30,31], respectively. Serum high-density lipoprotein (HDL-C) concentration was assayed using the colorimetric enzymatic method of [32]. Serum low-density lipoprotein (LDL-C) concentration was calculated according to the equation described by [33]. Serum Aspartate Aminotransferase (AST) and Alanine aminotransferase (ALT) activities were measured according to the colorimetric kit technique using Diamond Diagnostics co., Cairo, Egypt [34]. Serum Alkaline phosphatase (ALP) activities were measured according to the method described by [35]. The serum gamma-glutamyltransferase (GGT) activity was determined according to the process of [36]. The total bilirubin level in the serum was determined using kits purchased from Diamond Diagnostics, Co., Cairo, Egypt and was measured by a colorimetric method [37]. Serum total protein (TP) contents were estimated, as described by [38]. Serum albumin (Alb) content was determined according to [39], using a kit obtained from Diamond Diagnostics, co., Cairo, Egypt.

### 2.6. Analysis of the Antioxidant Status in Hepatic Tissues

The liver homogenate malondialdehyde (MDA) content was determined according to the colorimetric technique of [40], liver nitric oxide (NO) activity was estimated by the method of [41], liver homogenate H_2_O_2_ activity was assayed by the method of [42], and liver homogenates glutathione (GSH) and glutathione S transferase (GST) according to [43,44], respectively. Liver superoxide dismutase (SOD) and catalase (CAT) activity were estimated according to [45,46], respectively. All assays were evaluated using kits from Biodiagnostic Co. Dokki, Giza, Egypt.

### 2.7. Flowcytometric Analysis for B-Cell Lymphoma 2 (BCL-2), P53, BCL-2-like Protein 4 (Bax), and Caspase-3

The BCL-2, P53, Bax, and caspase-3 were assessed using a flow cytometer instrument (San Jose, CA, USA) in the Mansoura Children Hospital FACS calibur flow cytometer (Becton Dickinson, San Jose, CA, USA) [47], in which samples from the liver were prepared according to the method done by [48], with some modifications. In brief, the tissue’s fresh specimens were washed with isotonic tris EDTA buffer, centrifuged at 1800 rpm for 10 min, and then, the supernatant was removed. The liver samples were suspended in phosphate-buffered saline (PBS) with bovine serum albumin (BSA) divided into aliquots, fixed in ice-cold 96–100% ethanol stored at 4 °C for analyses. For Bcl-2, Bax, and Tumor protein p53 (P53), anti-B-cell lymphoma 2 (Bcl2), anti-Bax, and anti-P53 (Santa Cruz Biotechnology, Inc., Dallas, TX, USA.) were added to the PBS/BSA buffer and incubated for 30 min (diluted at 1:100), centrifuged at 400× *g* for 5 min for resuspending in 0.5% paraformaldehyde in PBS/BSA, and analyzed using a flow cytometer. Caspase-3 was done by using the following antibodies (Fluorescein isothiocyanate (FITC) rabbit anti-active caspase-3 (1:500) (CPP32, Yama, Apopain; BD Bioscience)). The analysis of the flow cytometer was carried out by using BD Accuri™ C6 (BD Biosciences, San Jose, CA, USA) with the Cell Quest Pro software (Becton Dickinson, San Jose, CA, USA) for data acquisition and analysis [49].

### 2.8. Determination of Hepatic Reactive Oxygen Species (ROS) and Interleukin 6 (IL-6) Content Using ELISA

ROS content was determined using the ROS kit from AMSBIO Co., Milton, UK, according to [50]. The IL-6 levels in the tissue were estimated quantitatively using a RAT ELISA kit from Ray Biotech, Inc. (Norcross, GA, USA).

### 2.9. Gene Expression Analysis

In 1 mL QIAzol (79306, QIAGEN Inc., Valencia, CA, USA), the total RNA content was extracted with chloroform from liver tissue. The corresponding cDNA was synthesized with RevertAid Premium reverse transcriptase (EP0733, Thermo Fisher Scientific, Darmstadt, Germany). Amplification curves and cycle threshold (CT) values were developed with Stratagene MX3005P software. The CTs of each sample were compared to the positive control group by the "alternative to CT" approach to estimate the RNA differences in the sample gene expressions. Primer sequence and information for relevant genes are summarized in Table 1.

### 2.10. Histopathological Studies

Liver tissues were accurately fixed in a neutral formalin solution (10%). They were dehydrated in an ascending series of ethanol, were cleared in xylene, were embedded in paraffin wax, and were sectioned at 5–7 μm by microtome and were stained with eosin and hematoxylin. The stained sections were examined and were photographed under a light microscope to detect histopathological changes [55].

### 2.11. Statistical Analysis

All data were expressed as means ± SE using one-way ANOVA, followed by Tukey’s multi-range, post-hoc check using SPSS software, version 20.0 (SPSS Inc., Chicago, IL, USA). Repeated measures ANOVA was used for determining a change in body weight at different intervals. The differences were statistically significant at *p* ≤ 0.05.

## 3. Results

### 3.1. Body Weight

**Figure 2:**
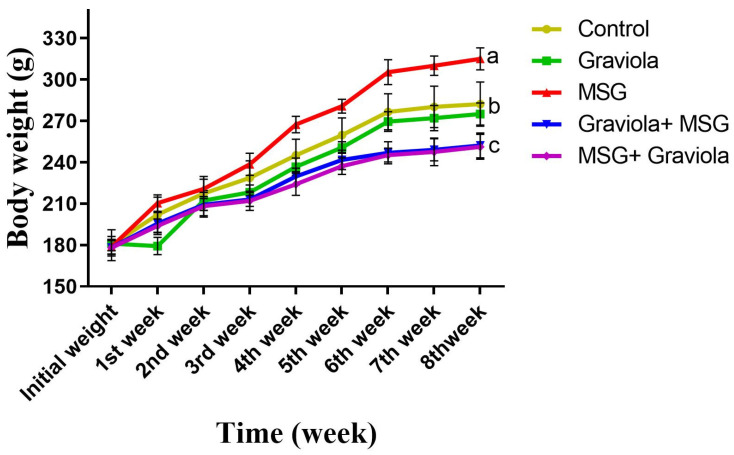
Body weight in control and differently treated rats. Data presented as means ± SEM for six rats in each group and % of change. Different superscript letters (a, b and c) indicate significant differences. The significant change at *p* ≤ 0.05. (N = 6).

At the end of the experiment (56 days), body weight was found to increase significantly in the MSG-treated group compared to the control group (*p* < 0.05). There was a significant decrease in body weight in rats orally administered MSG + GE and GE + MSG compared to the MSG-treated group.Concerning control one, there were no significant shifts with the Graviola treatment. (Figure 2).

### 3.2. Hematological and Biochemical Findings 

As shown in Table 2, the obtained result demonstrated that Hemoglobin (Hb), Red blood cells (RBCs), White blood cells (WBCs), Packed cell volume (PCV%), and platelets significantly decreased in the MSG-treated group compared to the control and Graviola groups. Graviola-treated groups, either protected or prophylactic, showed a significant increase in Hb, RBCs, WBCs, PCV%, and platelets related to the MSG-treated group.

The level of total cholesterol, triglyceride, and Low-density lipoprotein-cholesterol (LDL-C) and the liver enzymes Alanine aminotransferase (ALT). Aspartate aminotransferase (AST), Alkaline phosphatase (ALP).and gamma-glutamyltransferase (GGT) and the total bilirubin in the MSG-treated group were higher than that of the control group and Graviola group. In the same context, the Graviola-treated groups, either prophylactic or treated, showed significant decreases in these parameters. On the contrary, the levels of HDL-c, albumin, and total protein were significantly decreased (*p* < 0.05) in MSG-treated rats, which dramatically improved with Graviola treatment, as described in Table 3.

### 3.3. Hepatic Antioxidant Status

We examined the effects of the Graviola treatment on the MSG-induced hepatic injury. MSG’s toxicity hepatic toxicity through ROS formation encourages us to study Graviola’s antioxidant activity concerning MSG hepatic toxicity. The obtained data showed a significant decrease in hepatic GSH, GST, SOD, and CAT levels in the MSG group compared to the control group and Graviola group (*p* < 0.05), while the oral administration of GE to rat groups (MSG + GR and GR + MSG) resulted in a significant increase in GSH content than the MSG group, as shown in Table 4.

We showed a significant increase in MDA, H_2_O_2_, NO, and ROS concentrations in the MSG group compared to our data’s control group. Furthermore, the oral administration of GE (200 mg/kg BW) to the rat groups (MSG + Graviola and Graviola + MSG) resulted in a significant decrease in MDA concentration compared to the MSG group. Additionally, GE’s administration only showed no substantial change than the control group, as shown in Table 4.

### 3.4. Effect of Graviola on MSG-Induced Liver Cell Apoptosis

**Figure 3:**
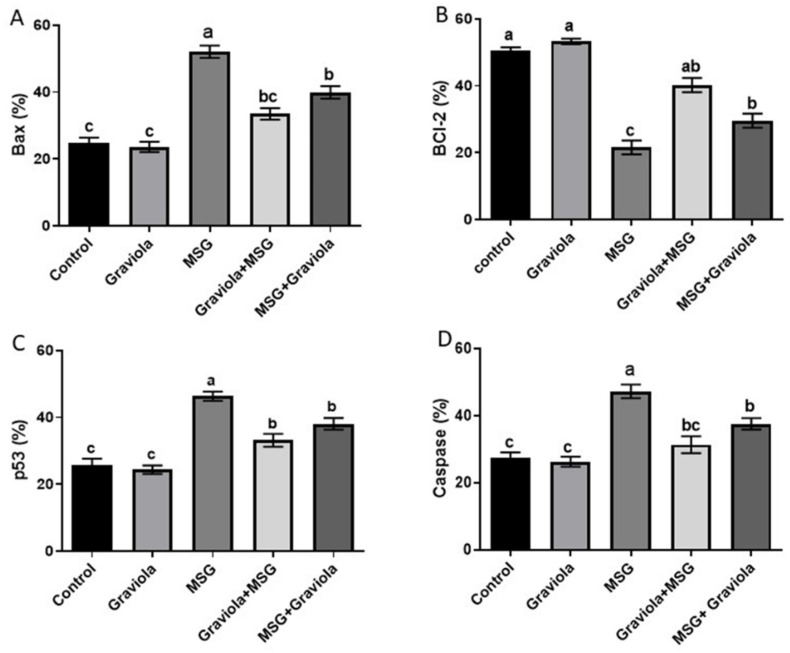
Flowcytometric analysis of the hepatic level of proapoptotic protein BCL-2-like protein 4 (Bax) (**A**), antiapoptotic protein B-cell lymphoma 2 (Bcl-2) (**B**), P53 (**C**), and caspase-3 (**D**) of different treated groups. Data are presented as means ± SE for six rats in each group and % of change. Different superscript letters (a, b and c) indicate significant differences. The significant change was at *p* ≤ 0.05.

Flow cytometric analysis was conducted to examine Graviola’s antiapoptotic effects on apoptosis in liver cells induced by MSG. The p53, caspase-3, and Bax levels of the MSG group were significantly higher than the control group (*p* < 0.05). Besides, the oral administration of GE (200 mg/kg BW) to the rat groups MSG + GE and GE + MSG resulted in a significant decrease in their expression levels compared to the MSG group. Additionally, GE administration showed no statistically significant difference from the control group, as shown in Figure 3. Conversely, the Graviola-treated group showed normalization to the BCL-2 level, which was significantly decreased by MSG administration. Please see the Figures S1–S4.

### 3.5. Effect of Graviola on the Levels of ROS and IL-6

**Figure 4:**
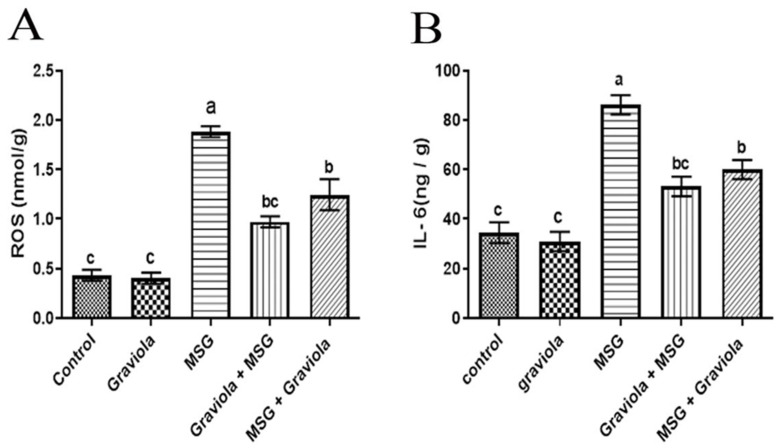
Hepatic ROS level (**A**) and interleukin 6 (IL-6) (**B**) of different treated groups. Data are presented as means ± SEM for six rats in each group and % of change. Different superscript letters (a, b and c) indicate significant differences. The significant change was at *p* ≤ 0.05.

There was a significant increase (*p* < 0.05) in ROS and IL-6 levels in the MSG group compared to the control group. Additionally, both MSG + GE and GE + MSG showed a significant decrease in ROS and IL-6 levels, whereas the Graviola administration alone had no significant difference from the control group, as shown in Figure 4.

### 3.6. Effect of Graviola on the Histopathological Alteration Induced by MSG in Liver

**Figure 5:**
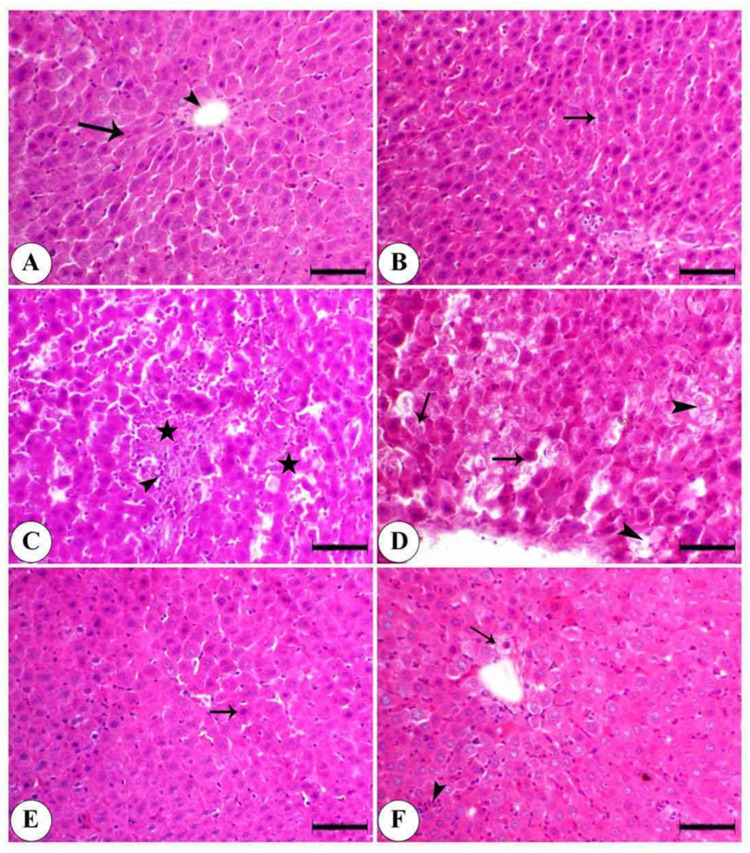
Photomicrograph of the liver of the control and differently treated groups of animals. (**A**) Control showing normal hepatocytes (arrow) arranged in cords around the central vein (arrowhead). (**B**) Graviola is showing normal hepatocytes arranged in cords separated by blood sinusoids (arrow). (**C**) MSG showing periportal hepatic necrosis (astars) associated with mononuclear cell infiltration (arrowhead). (**D**) MSG is showing single-cell necrosis (arrow) with a loss of cellular details and nuclei of some hepatocytes (arrowhead). (**E**) Graviola + MSG is showing a few pyknotic nuclei (arrow) and a mild degree of hepatocyte degeneration (arrowhead). (**F**) MSG + Graviola showing limited centrilobular hepatic vacuolation (arrow) and mononuclear cell infiltration (arrowhead). Stained with hematoxylin and eosin (H & E), scale bar = 50 µm.

As shown in Figure 5, the control showed normal hepatocytes arranged in cords around the central vein. The Graviola-treated group alone, showing normal hepatocytes arranged in cords separated by blood sinusoid MSG, showed periportal hepatic necrosis associated with mononuclear cells infiltration and hepatic vacuolation, (arrowhead) single-cell necrosis, and a loss of cellular details and nuclei of some hepatocytes. The Graviola + MSG (prophylactic group) showed a few pyknotic nuclei of hepatocytes and a mild degree of hepatocyte degeneration. In the MSG + Graviola, limited centrilobular hepatic vacuolation and mononuclear cell infiltration were observed.

### 3.7. Effect of Graviola on Silent Information Regulator Protein One (SIRT1), Fatty Acid Synthase (FAS), and Inducible Nitric Oxide Synthase (iNOS) Gene Expression

**Figure 6:**
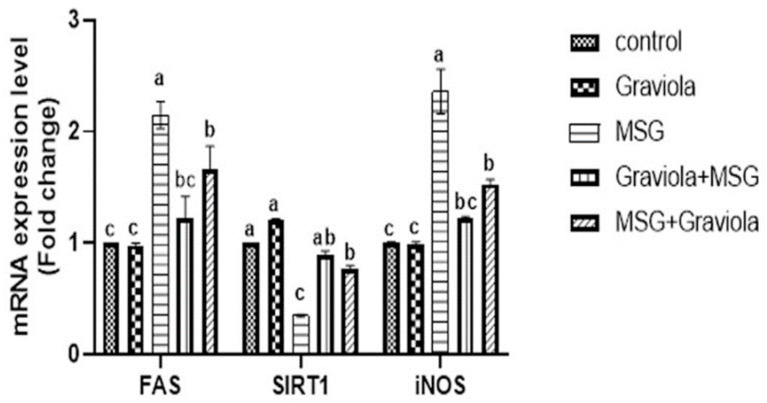
Expression of fold changes of hepatic SIRT1, silent information regulator protein one gene expression; FAS, fatty acid synthase; and iNOS, inducible nitric oxide synthase. Data were analyzed with one-way ANOVA, followed by Tukey’s multiple comparison test. Different superscript letters (a, b and c) indicate significant differences at *p* < 0.05. Error bars represent mean ± SEM, N = 6.

As shown in Figure 6, the relative ratio of hepatic SIRT1 gene expression was significantly reduced in the MSG-treated group, and the comparable rate of SIRT1 gene expression was improved considerably in Graviola-treated rats. FAS gene expression with MSG treatment was upregulated considerably, showing significant downregulation with the Graviola treatment. Compared to the control group, the relative expression of the hepatic iNOS in rats treated with MSG gene expressions were markedly upregulated and significantly downregulated in rats treated with Graviola compared to rats treated with MSG.

## 4. Discussion

Currently, there is a substantial increase in the use of food additives. Organic compounds are deliberately added to food in small quantities during the production process to improve the organoleptic quality of foods, such as flavor, color, taste, appearance, and texture [3]. Food additives can instantly be harmful or be long-lasting if they are continuously consumed. Immediate effects may include headaches, changes in energy levels, mental concentration and behavior changes, and immune response [56]. One of those food additives widely used as an enhancer of flavor is monosodium glutamate (MSG). It is a glutamic acid salt amino acid [1]. This encourages us to search for a natural food additive, such as Graviola, that could offset the oxidative and inflammatory processes induced by MSG in hepatic tissues. Our data revealed that MSG causes a significant increase in body weight. This increase may be attributed to the fact that MSG can improve foods’ palatability by having a favorable effect on the appetite center [57] and enhancing the chemosensory perception [58]. Increased IL-6, resistin, and tumor necrosis factors in adipose tissues are the primary effects of MSG on body weight. The elevated serum levels of resistin and insulin can deteriorate the visceral adipose tissue [59]. Additionally, the ingestion of MSG has a local effect. It activates the celiac and gastric branches of the vagus nerve when found in the gastrointestinal tract, causing the activation of limbic, hypothalamus, insular cortex and nucleus tracts, and solitary tracts, which eat many foods [60]. An oral administration of Graviola normalized the body weight, and these findings are in agreement with [61,62,63]. The decrease in triglyceride and overall cholesterol is attributed to this, since Graviola has a hypolipidemic effect and hypolipidemic agents such as tannins, which reduce the cholesterol absorption and, consequently, reduce the body weight gain [64,65]. The lipid profile, including total cholesterol (TC), triglyceride (TG), and LDL-C, increased significantly in the serum of rats administered MSG. At the same time, the HDL-C content was reduced, as shown in Table 3; this result was inconsistence with [66,67], who reported that MSG could increase the activity of coenzyme A (HMG CoA) reductase, 3-hydroxyl-3-methylglutaryl, the limiting factor of cholesterol biosynthesis, which results in increased cholesterol synthesis and hyperlipidemia, with increased serum TG and TC, shifting the glucose metabolism towards lipogenesis. The prophylactic and protective roles of orally administered Graviola in normalized parameters of the lipid profile, as shown in Table 3, could be attributed to the involvement of hypolipidemic agents in the GE [62,63]. The hypolipidemic and antioxidant effects of Graviola due to the presence of agents such as tannins and other polyphenolic compounds cause decreasing cholesterol absorption by deactivating coenzyme-A (HMG-CoA) reductase hydroxymethylglutaryl [65]. Our findings showed that MSG can significantly increase the liver enzymes ALT, AST, ALP, and GGT due to the cytotoxic effect of MSG, which resulted in damage to liver cells and canaculae and the release of these enzymes in the circulation [68]. Moreover, MSG toxicity creates ammonium ions that cause hepatic toxicity through the formation of ROS that react with polyunsaturated fatty acids contained within cell membranes that cause plasma and mitochondrial membranes to deteriorate with the release of hepatic enzymes [69] via preserving the structural integrity of the hepatic cell membrane or regenerating damaged liver cells [70]. Graviola preserves and prevents the leakage of the intracellular enzyme70 liver injury triggered by MSG Graviola [71], which supports our finding of the protective role of Graviola. Our research reaffirmed that MSG has harmful effects on hematological parameters, with characteristic leukopenia consistent with [72,73] attributed to RBCs’ short half-life due to the hemolytic effect of MSG. This may be due to the atrophy induced by MSG in the gastric mucosa (gastritis) as the L-form of glutamic acid is acidic, resulting in a reduction in intrinsic factor synthesis leading to vitamin B12 malabsorption, which is the main cause of anemia [74]. MSG caused leukopenia, which was in the same line as [75], who attributed this effect to the immune-suppressant effect of MSG due to MSG’s hazardous effects on the thymus and spleen. Graviola significantly cured the adverse impact of MSG. The obtained result was in the same line with [76], who attributed this finding to Graviola’s ability to restore body fluids and stimulate erythropoietin [77]. Orally administered MSG led to increased oxidative stress markers, such as MDA, ROS, NO, and H_2_O_2_, and decreased SOD, CAT, GST, and GSH that supported this finding by [9,68,78,79,80,81] as a result of the exhaustion of SOD and accumulation of H_2_O_2_ as a result of ROS formation as a result of MSG. Besides [9], this effect is revealed to MSG lipogenesis characters that consume nicotinamide adenine dinucleotide (NAD) + hydrogen (H) (NADH). Similarly, the conversion of the majority of GSH in the liver to glutathione disulfide (GSSG) by the glutathione reductase enzyme to protect the liver cells from toxic material damage decreased the GSH level. The elevated levels of MDA and NO return to the difficulty of glutamate transportation across the cell membrane, which initiate lipid peroxidation (LPO) and alter the cell redox state [82], leading to membrane damage [83]. Graviola administration normalized the oxidant status of the liver cells, this result being in harmony with [43] due to Graviola antioxidant activity [84]. Graviola has a protective role against free radicals (OH) and H_2_O_2_ [85]. Therefore, it stopped the elevation of LPO [86] and converted the ROS to nontoxic or dangerous goods [87]. Graviola possesses potent antioxidant properties due to the presence of acetogenins, which can play an essential and significant role in free radical scavenging [85]. The IL-6 proinflammatory protein is one of the families of cytokines that help organisms react to infectious agents and increases the development of Interleukin-6 (IL6) inflammation boost in the MSG community due to chronic inflammation leading to overexpression of the IL6 mRNA gene [88]. Our results showed that the Graviola extract normalized the level of IL6 that is inconsistent with [89] due to the presence of anti-inflammatory agents in Graviola extracts, such as alkaloids, saponins, flavonoids, and tannins, which inhibit prostaglandin synthesis [90,91]. This study shows that the administration of MSG led to significant increases in P53, caspase-3, and apoptotic (Bax) proteins and a substantial reduction in antiapoptotic (Bcl-2) proteins. This was in the same line with [92,93], who explained that glutamate-induced the Ca2^+^ influx and destruction of the internal mitochondrial membrane potential, resulting in the unregulated mitochondrial permeability of the pores to apoptotic markers [94]. Graviola was found to decrease the higher levels of Bax, caspase 3, and P53 and significantly increase Bcl2 in MSG-treated rats. This finding was in agreement with [95] on the overexpression of bcl2, which prevents DNA fragmentation due to its antioxidant activity and blocks the cytochrome C release and mitochondrial permeability.

In terms of the current histopathological findings, normal hepatocytes arranged in regulated cords around the central vein and GE-treated group, and periportal hepatic necrosis associated with mononuclear cell infiltration, hepatic vacuolation was seen in MSG-treated rats. Such findings are comparable to [96,97,98] findings. Due to MSG, therefore, the cell is not able and cannot repair the damage entirely due to excess glutamine. Vesicular degeneration and necrosis are expected to occur in hepatic tissue [99]. Increased central adiposity and the gene expression of white adipose tissue can also cause fatty liver damage caused by hepatic exacerbation.

Furthermore, MSG has been shown to cause oxidative stress and hepatotoxicity [100]. The vacuolization of hepatocytes has been described as a ballooning degeneration. It has been interpreted as a cellular defense mechanism for harmful substances, which collects and prevents interference with the biological acting elements. Additionally, the high level of MDA caused by the LPO effect of MSG may lead to hepatic necrosis [68]. On the other hand, the administration of GE had a therapeutic effect on the liver architecture for rats before and after MSG treatment. Such findings are comparable to those of [101]. The involvement inhibits cyclo-oxygenase 2 of tannins in GE and acetogenin [102,103].

**Figure 7:**
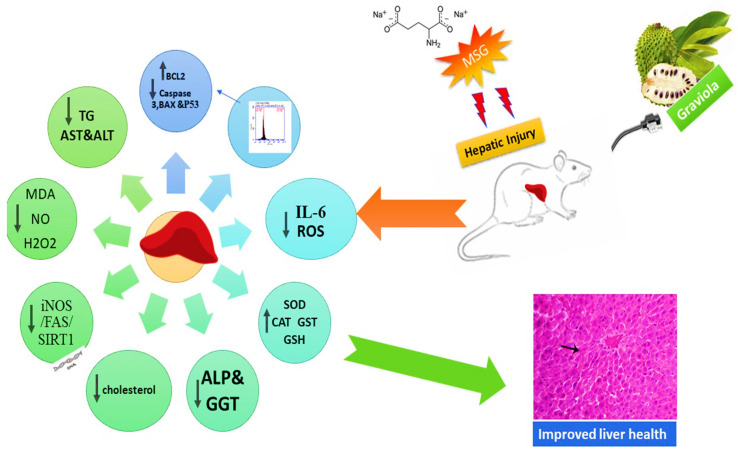
Graphical summary showing the effect of Graviola extract on MSG-induced hepatic injuries.

Sirt1 is a Sirtuin family prototype. It is a crucial metabolism regulator involved in cell metabolism, fat utilization and insulin tolerance, cell division and senescence, metabolic stress, and disease [104]. Liver Sirt1 appears to be playing a significant function in the regulation of homeostasis. However, fatty acid beta-oxidation and gluconeogenesis are reduced by its depletion. The upregulation expression of SIRT1 with Graviola could be due to its antioxidant action, which could explain Graviola’s anti-inflammatory pathway as a regulated SIRT1 activation of various factors, including the transcription factor nuclear factor kappa B (NF-κB) [105]; therefore, it could regulate the inflammation process. On the contrary, FAS gene expression was significantly upregulated with MSG treatment, which showed a marked downregulation with the Graviola treatment. This result was supported by [106] by finding that MSG rats are dyslipidemic, counteracting the stimulated liver lipogenic process by the high expression of their master regulator genes, Sterol regulatory element-binding transcription factor 1 (SREBP1c) target genes and fatty acid synthase (FAS) [107]. They showed that MSG rats developed an insulin-resistant state and increased oxidative stress, and severe liver injury characterized by inflammation and metabolic signs involved lipogenesis. This supports our obtained results for the body weight. Conversely, the NO levels in the MSG rats were also boosted in conjunction with a substantial increase in gene expression of the iNOS (the source of NO) in the current study. NO and iNOS perform complicated roles in the development of hepatic injury. The relationship between NO and hepatic injury includes cytotoxicity, the inflammatory response, and metabolic energy abnormality. An extreme extracellular expression of iNOS induced an outrageous leak of NO to cells with developed adverse effects [108]. Therefore, the hepatic expression of iNOS in different models for liver injury is essential for hepatic repair [109]. As shown in Figure 7, Graviola supplementation can overcome the monosodium glutamate-induced hepatic injury through many intarcellular pathways.

## 5. Conclusions

Food additives, especially monosodium glutamate, induce hepatic injury in rats. Graviola supplementations overcame these alterations by modulating liver apoptosis markers and enhancing the hepatic antioxidant status, which was accompanied by a reduction in inflammatory markers and cellular apoptosis. Additionally, by modulating the lipogenesis gene, Graviola improved the transcriptomic effect induced by monosodium glutamate. Clinical human trials are required to validate the animal studies to qualify this effect found in hepatic rats.

## Figures and Tables

**Table 1:** Primers sequences.

Genes	5′–3′ Primer Sequence	Accession Number	References
FAS	F: CCTGGACAACATGGTAGCTGC	NM 017332.1	[51]
	R: GCAGTGCCTTCCTTGAGAACAG		
SIRT1	F: TGA CTT CAG ATC AAG AGA TGG TAT TTA TG	NM 001372090	[52]
	R: TGG CTT GAG GAT CTG GGA GAT		
iNOS	F: GGATATCTTCGGTGCGGTCTT	S71597	[53]
	R: CTGTAACTCTTCTGGGTGTCAGA		
GAPDH	F: TCAAGAAGGTGGTGAAGCAG	NM 017008.4	[54]
	R: AGGTGGAAGAATGGGAGTTG		

SIRT1, silent information regulator protein one gene expression; iNOS, inducible nitric oxide synthase; FAS, fatty acid synthase; *GAPDH*, glyceraldehyde 3-phosphate dehydrogenase; and GGT, gamma-glutamyltransferase.

**Table 2:** Effects of Graviola and monosodium glutamate (MSG) on the hematological parameters.

Parameters	Control	Graviola	MSG	Graviola + MSG	MSG + Graviola
Hb(g/L)	154.6 ± 1.35 ^a^	159.2 ± 0.45 ^a^	85 ± 0.64 ^c^	115.6 ± 0.78 ^b^	105.4 ± 0.62 ^b^
RBCs (10^12^/L)	9.22 ± 0.56 ^a^	9.27 ± 0.49 ^a^	4.06 ± 0.26 ^c^	6.00 ± 0.30 ^b^	5.93 ± 0.46 ^b^
PCV(L/L)	0.510 ± 0.01 ^a^	0.525 ± 0.01 ^a^	0.280 ± 0.01 ^c^	0.381 ± 0.06 ^b^	0.347 ± 0.07 ^b^
WBCs (10^9^/L)	9.50 ± 0.31 ^a^	10.20 ± 0.33 ^a^	5.45 ± 0.32 ^c^	7.25 ± 0.31 ^b^	7.07 ± 0.34 ^b^
Lymphocyte%	73.12 ± 1.15 ^a^	74.20±1.10 ^a^	64.92 ± 1.19 ^c^	67.00 ± 1.7 ^b^	68.00 ± 1.7 ^b^
Neutrophil%	18.0 ± 1.3 ^a^	18.1 ± 1.9 ^a^	7.0 ± 1.1 ^c^	16.4 ± 1.8 ^b^	16.9 ± 1.1 ^b^
Platelets (10^9^/L)	755.9 ± 35.38 ^a^	764.3 ± 40.90 ^a^	269.6 ± 25.23 ^c^	458.4 ± 46.40 ^b^	443.3 ± 44.20 ^b^

Data presented as means ± SEM for six rats in each group and % of change. The significant change was at *p* ≤ 0.05. Different superscript letters (a, b and c) indicate significant differences in the same column (N = 6). Hemoglobin (Hb), Red blood cells (RBCs), Packed cell volume (PCV), White blood cells (WBCs).

**Table 3:** Effect of Graviola and MSG on the serum biochemical parameters.

Parameters	Control	Graviola	MSG	Graviola + MSG	MSG + Graviola
Total cholesterol (mmol/L)	2.8 ± 0.06 ^c^	2.76 ± 0.01 ^c^	3.25 ± 0.1 ^a^	3.06 ± 0.1 ^b^	3.09 ± 0.2 ^b^
Triglycerides (mmol/L)	1.10 ± 0.03 ^c^	1.07 ± 0.02 ^c^	1.46 ± 0.04 ^a^	1.29 ± 0.01 ^b^	1.32 ± 0.02 ^b^
LDL-C (mmol/L)	0.85 ± 0.01 ^c^	0.87 ± 0.02 ^c^	1.88 ± 0.07 ^a^	1.46 ± 0.06 ^b^	1.43 ± 0.0.04 ^b^
HDL-C (mmol/L)	1.30 ± 0.06 ^a^	1.32 ± 0.01 ^a^	0.796 ± 0.02 ^c^	1.15 ± 0.02 ^b^	1.097 ± 0.02^b^
ALT (µkat/L)	0.51 ± 0.008 ^c^	0.48 ± 0.01 ^c^	0.99 ± 0.03 ^a^	0.73 ± 0.01 ^b^	0.75 ± 0.01 ^b^
AST (µkat/L)	0.46 ± 0.008 ^c^	0.49 ± 0.006 ^c^	1.1 ± 0.08 ^a^	0.72 ± 0.01 ^b^	0.79 ± 0.009 ^b^
ALP (µkat/L)	2.19 ± 0.14 ^c^	2.16 ± 0.02 ^c^	2.83 ± 0.02 ^a^	2.50 ± 0.06 ^b^	2.48 ± 0.02 ^b^
GGT (µkat/L)	0.336 ± 0.005 ^c^	0.334 ± 0.008 ^c^	0.475 ± 0.003 ^a^	0.416 ± 0.004 ^b^	0.423 ± 0.004 ^b^
TB (µmol/L)	7.70 ± 0.21 ^c^	6.84 ± 0.18 ^c^	21.72 ± 0.79 ^a^	13.0 ± 0.64 ^b^	13.17 ± 0.26 ^b^
Albumin (g/L)	38 ± 0.64 ^a^	38.5 ± 0.87 ^a^	20 ± 0.85 ^c^	32 ± 1.25 ^b^	30 ± 1.31 ^a,b^
Total proteins (g/L)	125.90 ± 0.65 ^a^	127.90 ± 0.86 ^a^	84.2 ± 1.49 ^c^	103 ± 2.17 ^a,b^	100.00 ± 2.10 ^a,b^

Data presented as means ± SEM for (6) rats in each group and % of change. The significant change was at *p* ≤ 0.05. Different superscript letters (a, b and c) indicate significant differences in the same column (N = 6). Serum gamma-glutamyltransferase (GGT). High-density lipoproteins (HDL-C). Low-density lipoproteins (LDL-C). Serum total bilirubin (TB). Alanine aminotransferase (ALT). Aspartate aminotransferase (AST), Alkaline phosphatase (ALP).

**Table 4:** Effects of Graviola and MSG extract on liver oxidative status of different rat groups.

Parameters	Control	Graviola	MSG	Graviola + MSG	MSG + Graviola
MDA (nmol/g)	685.8 ± 36.30 ^c^	678.2 ± 36.31 ^c^	1216 ± 18.01 ^a^	800.4 ± 18.88 ^b^	815.8 ± 18.80 ^b^
NO (μ mol/g)	18.23 ± 0.52 ^c^	18.11 ± 0.50 ^c^	35.48 ± 0.54 ^a^	25.00 ± 0.50 ^b^	26.10 ± 0.55 ^b^
H_2_O_2_ (mM/g)	1.32 ± 0.10 ^c^	1.28 ± 0.12 ^c^	4.83 ± 0.15 ^a^	3.30c ± 0.10 ^b^	3.37 ± 0.11 ^b^
SOD (U/g)	92.09 ± 2.77 ^a^	95.31 ± 2.40 ^a^	59.28 ± 2.56 ^c^	79.48 ± 2.66 ^b^	75.84 ± 2.54 ^b^
CAT (U/g)	189.5 ± 4.40 ^a^	192.0 ± 2.89 ^a^	128.5 ± 4.06 ^d^	170.6 ± 4.66 ^c^	165.4 ± 4.54 ^b^
GST (U/g)	5.40 ± 0.57 ^a^	5.87 ± 0.57 ^a^	1.25 ± 0.22 ^c^	3.24 ± 0.40 ^b^	3.06 ± 0.24 ^b^
GSH (mmol/g)	5.50 ± 0.24 ^a^	5.54 ± 0.29 ^a^	2.86 ± 0.17 ^c^	4.20 ± 0.16 ^b^	4.00 ± 0.15 ^b^

Data presented as means ± SEM for six rats in each group and % of change. The significant change at *p* ≤ 0.05 (N = 6). Different superscript letters (a, b, c and d) indicate significant differences in the same column. Malondialdehyde (MDA), nitric oxide (NO), glutathione (GSH), glutathione S transferase (GST), superoxide dismutase (SOD), and catalase (CAT).

## Supplementary Materials

The following are available online at https://www.mdpi.com/2076-2615/10/11/1996/s1, Figure S1: Bax flowcytometry, Figure S2: Bcl2 flowcytometry, Figure S3: Caspase 3 flowcytometry, Figure S4: P53 flowcytometry.

## Abbreviation

ALB	Serum Albumin
BAX	BCL-2-like protein 4
BCL2	B-cell lymphoma 2
CAT	Catalase
FAS	Fatty acid synthase
GAPDH	Glyceraldehyde-3-phosphate dehydrogenase
GSH	Reduced glutathione
GST	Glutathione S transferase
GGT	Serum gamma-glutamyl transferase
H_2_O_2_	Hydrogen peroxide
HDL-C	High-density Lipoprotein
IL-6	Interleukin 6
NOS	Nitric oxide synthase
iNOS	inducible nitric oxide synthase
LDL-C	Low-Density Lipoprotein
MDA	Malondialdehyde
MSG	Monosodium glutamate
NO	Nitric oxide
ROS	Reactive oxygen species
SIRT1	Silent information regulator protein one gene expression
SOD	Superoxide dismutase
TB	Serum total bilirubin
TP	Serum Total Protein
p53	Tumor protein
