# Supplementary material for: Ameliorative Effect of Graviola (Annona muricata) on Mono Sodium Glutamate-Induced Hepatic Injury in Rats: Antioxidant, Apoptotic, Anti-inflammatory, Lipogenesis Markers, and Histopathological Studies
Source: Animals (Basel). 2020 Oct 30;10(11):1996. doi: 10.3390/ani10111996 (PMC7693810; doi:10.3390/ani10111996)
Supplement: Supplementary file 1 [file animals-10-01996-s001.pdf]

animals

MDPI

Article

Ameliorative Effect of Graviola (*Annona muricata*) on Mono Sodium Glutamate-Induced Hepatic Injury in Rats: Antioxidant, Apoptotic, Anti-inflammatory, Lipogenesis Markers, and Histopathological Studies

Mustafa Shukry [1,*] Ahmed M. El-Shehawi [2,3], Wafaa M. El-Kholy [4], Rasha A. Elsisy [5], Hazem S. Hamoda [6], Hossam G. Tohamy [7], Mohamed M. Abumandour [8] and Foad A. Farrag [9]

1 Department of Physiology, Faculty of Veterinary Medicine, Kafrelsheikh University, 33511 Kafrelsheikh, Egypt
2 Department of Biotechnology, College of Science, Taif University, P.O. Box 11099, Taif 21944, Saudi Arabia; elshehawi@hotmail.com
3 Department of Genetics, Faculty of Agriculture, Alexandria University, 21527 Alexandria, Egypt
4 Department of Zoology, Faculty of Science, Mansoura University, 35516 Mansoura, Egypt; wafaa_elkholy2002@yahoo.com
5 Department of Anatomy, Faculty of Medicine, Kafrelsheikh University, 33516 Kafrelsheikh, Egypt; Rasha_2002@yahoo.com
6 Department of Anatomy and Embryology, Faculty of Veterinary Medicine, Aswan University, 81528 Aswan, Egypt; Hamouda2000@yahoo.com
7 Department of pathology., Faculty of Veterinary Medicine, Alexandria University, 22785 Alexandria, Egypt; hossam.gafar@yahoo.com
8 Department of Anatomy and Embryology., Faculty of Veterinary Medicine, Alexandria University, 22785 Alexandria, Egypt; m.abumandour@yahoo.com
9 Department of Anatomy and Embryology, Faculty of Veterinary Medicine, Kafrelsheikh University, 33511 Kafrelsheikh, Egypt; foad.farrag@yahoo.com
* Correspondence: mostafa.ataa@vet.kfs.edu.eg

Received: 29 September 2020; Accepted: 23 October 2020; Published: date

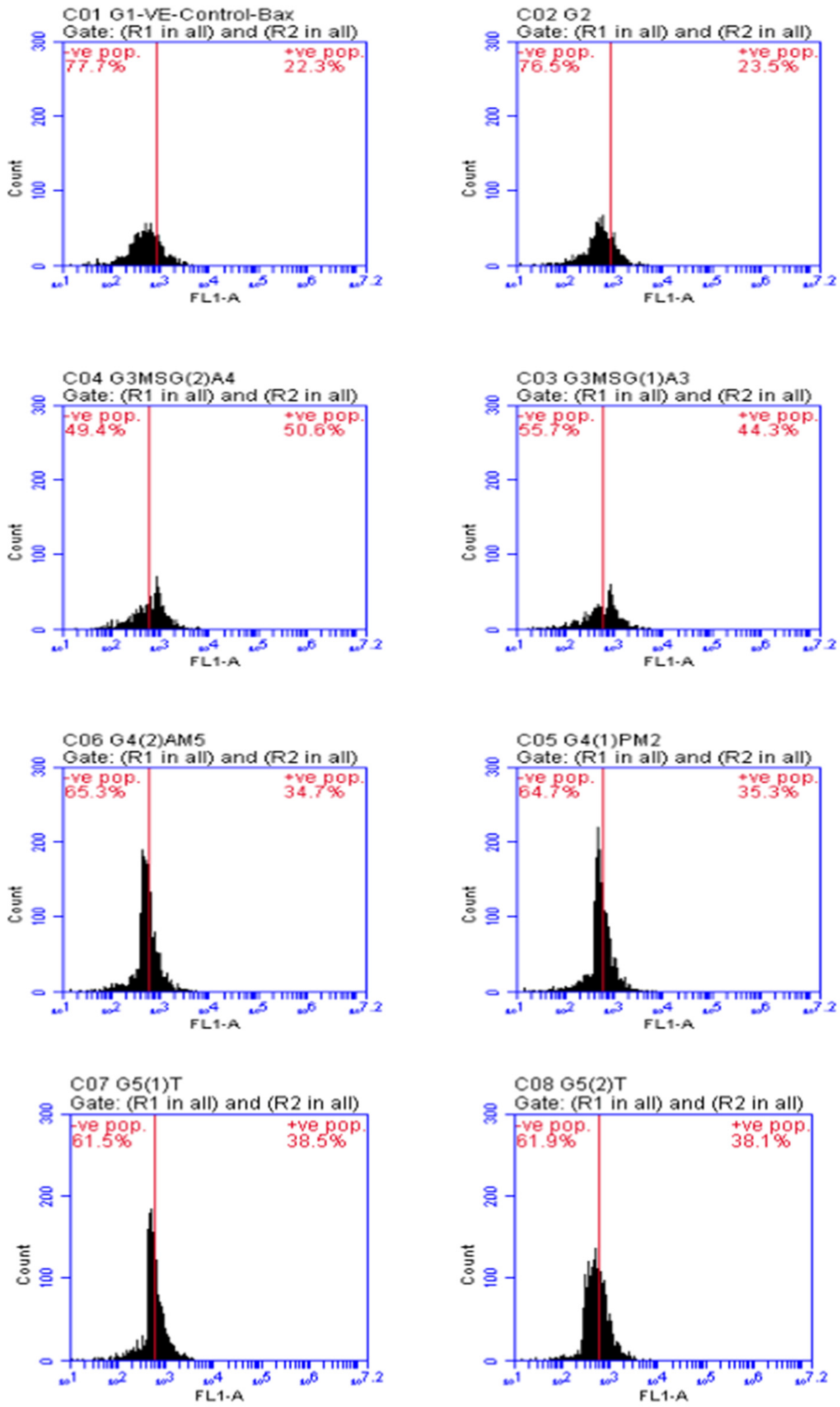

Figure S1: Bax flowcytometry.

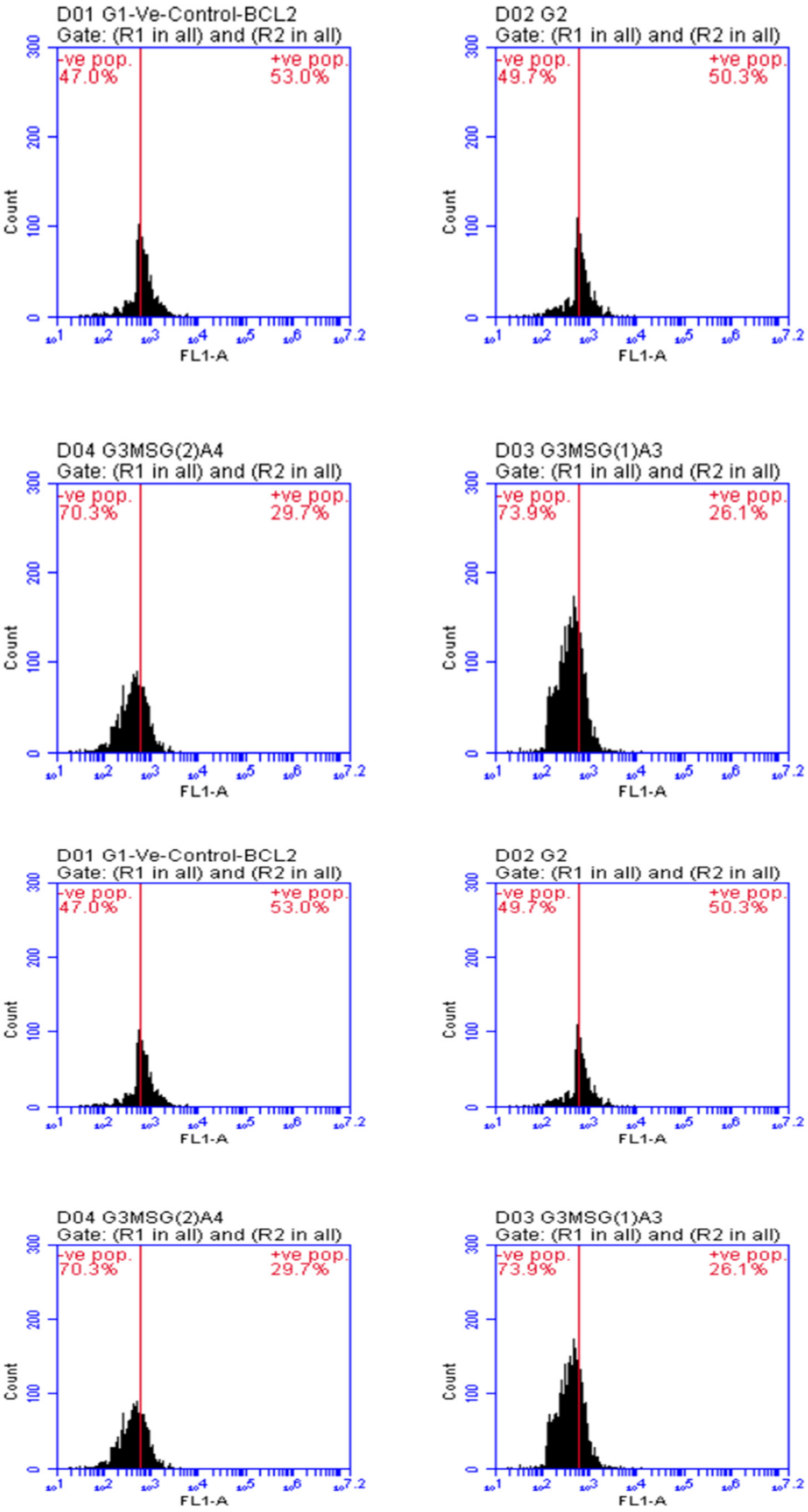

Figure S2: Bcl2 flowcytometry.

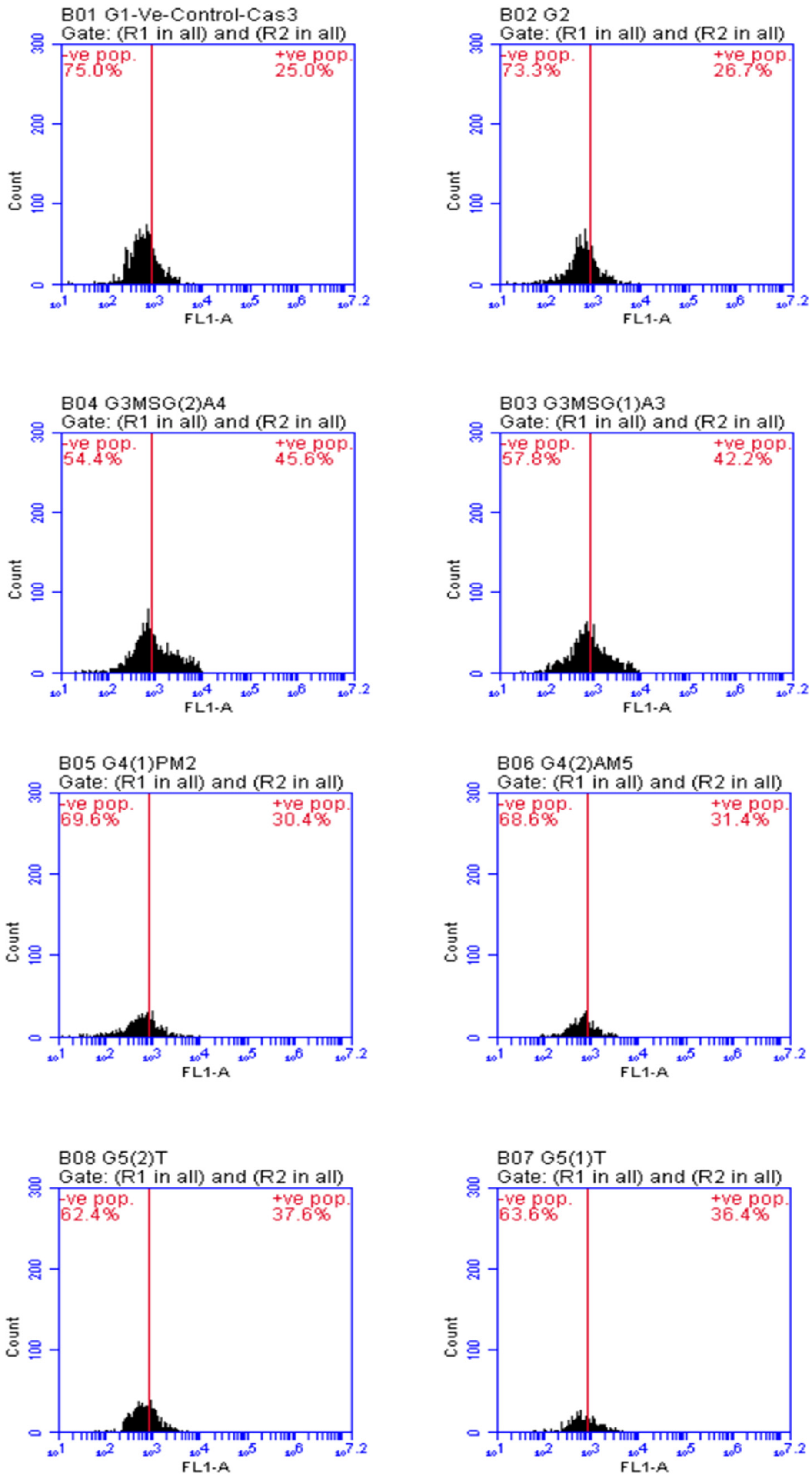

Figure S3: Caspase 3 flowcytometry.

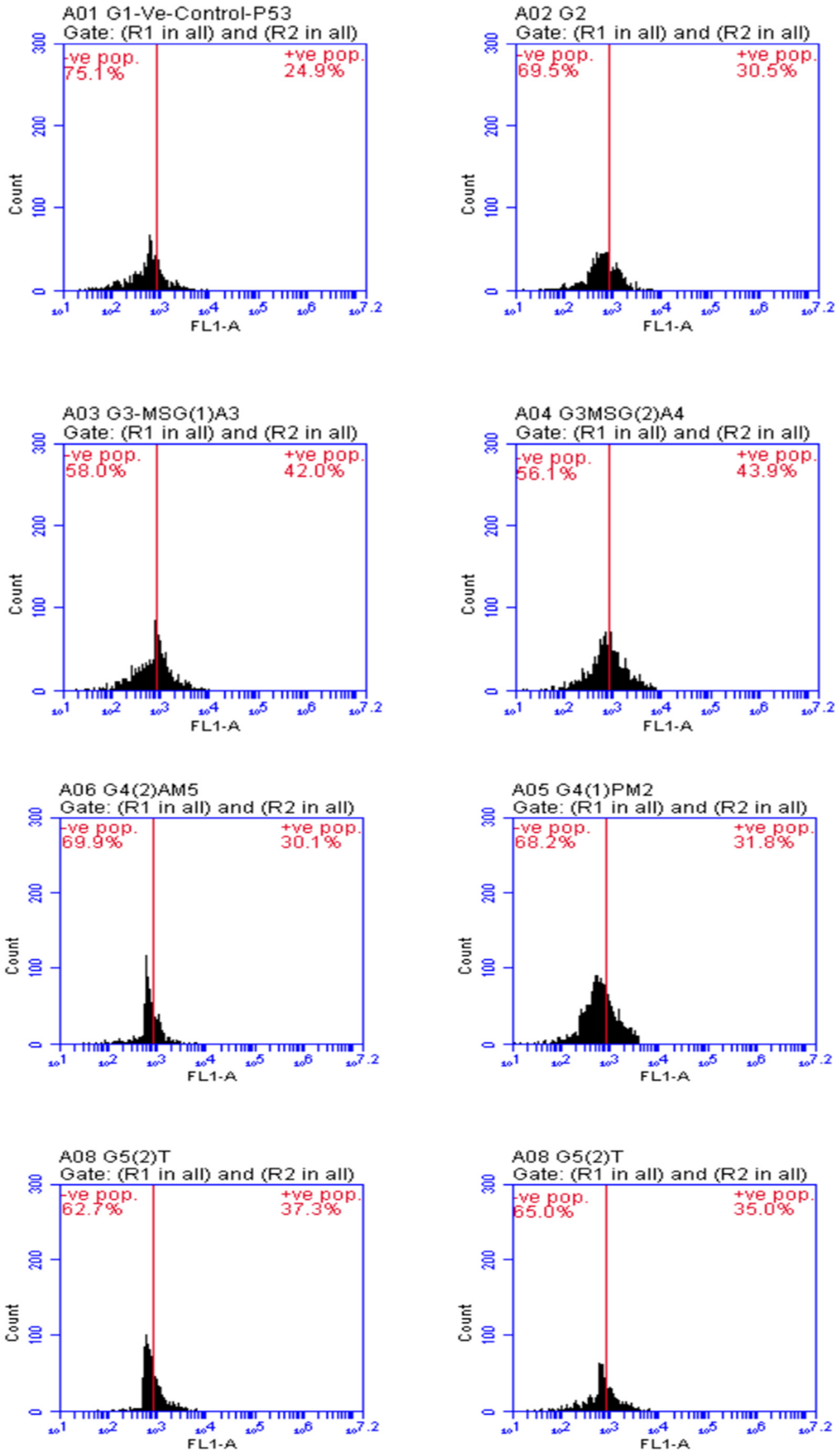

Figure S4: P53 flowcytometry.
